# ^1^H, ^13^C, ^15^N resonance assignment of the C-terminal domain of the bifunctional enzyme TraI of plasmid R1

**DOI:** 10.1007/s12104-018-9863-y

**Published:** 2019-01-07

**Authors:** Bhattiprolu Krishna, Nina Gubensäk, Gabriel E. Wagner, Ellen Zechner, Sandra Raffl, Walter Becker, Evelyne Schrank, Klaus Zangger

**Affiliations:** 1 Institute of Chemistry, University of Graz, 8010 Graz, Austria; 2 Institute of Hygiene, Microbiology and Environmental Medicine, Medical University of Graz, 8010 Graz, Austria; 3 Institute of Molecular Biosciences, University of Graz, BioTechMed-Graz, 8010 Graz, Austria

**Keywords:** NMR spectroscopy, Bacterial conjugation, TraI, Plasmid R1, Bifunctional enzyme

## Abstract

Transfer of genetic material is the main mechanism underlying the spread of antibiotic resistance and virulence factors within the bacterial community. Conjugation is one such process by which the genetic material is shared from one bacterium to another. The DNA substrate is processed and prepared for transfer by a multi-protein complex called the relaxosome .The relaxosome of plasmid R1 possesses the most crucial enzyme TraI which, both nicks and unwinds the dsDNA substrate. TraI comprises 1765 residues and multiple functional domains, including those catalyzing the DNA trans-esterase (relaxase) on the dsDNA designated for a conjugative transfer and DNA helicase activities. Structural and functional studies have been reported for most of the TraI except the C-terminal domain spanning from residue 1630 to 1765. This region is the least understood part of TraI and is thought to be highly disordered and flexible. This region, being intrinsically disordered, is hypothesized to be serving as an interacting platform for other proteins involved in this DNA transfer initiation mechanism. In this work, we report the ^1^H, ^13^C, ^15^N resonance assignment of this region as well as the secondary structure information based on the backbone chemical shifts.

## Biological background

Bacterial conjugation is a major mechanism driving rapid dissemination of genetic information among the bacterial community, leading to the spread of antibiotic resistance and virulence factors. This transfer is important for bacteria to adapt and evolve in variable environments. Transfer of DNA also takes place between the bacterial and eukaryotic communities (Christie et al. 2017; Ilangovan et al. 2015).The most studied conjugation system in Gram-negative bacteria is the F family of plasmids, which includes F, R1 and pED 208. The protein investigated in this work, TraI, is encoded by the R1 plasmid. Gram-negative bacteria utilize type IV secretion systems (T4SS) for a successful transfer of the conjugative plasmid from the donor to the recipient cell. In the donor, conjugation is initiated by three major components known as the relaxosome, transferosome and coupling proteins. The relaxosome is a multi-protein complex, which prepares the conjugative plasmid in the cytoplasm by nicking and unwinding it. The transferosome consists of the proteins, which are involved in the T4SS pilus formation and retraction, to make a close physical contact between donor and recipient cells. Finally, the coupling proteins ‘couple’ components of the relaxosome to the transferosome for a successful substrate transfer (Lawley et al. 2003).

**Fig. 1:**
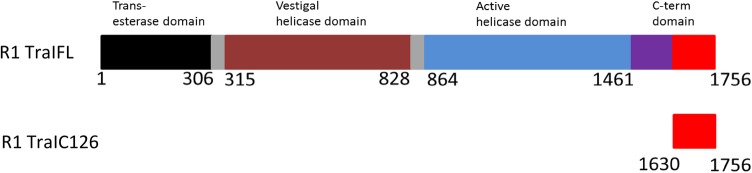
Structural motifs of TraI highlighting the far C-terminal domain (R1 TraIC126)

TraI of plasmid R1 is a multi-functional enzyme of the relaxosome that is essential for the conjugative transfer of F like plasmids (Wong et al. 2012; Lang et al. 2010, 2011; Haft et al. 2006). To specifically initiate the process of DNA transfer, R1 TraI first nicks a unique phosphodiester bond at *nic* site within the plasmid origin of transfer (*oriT*), binds covalently to the 5′ end of the nicked plasmid stand through a tyrosine residue and finally unwinds the nicked strand of plasmid DNA with the help of its functional helicase domain (Matson and Ragonese 2005). Recently a cryo-EM structure of the three functional domains of R1 TraI: the DNA trans-esterase, vestigial helicase and an active helicase, was published (Fig. 1). These authors found that R1 TraI dimerizes in the presence of DNA including the *oriT* sequence resulting in a well-coordinated function of trans-esterase and relaxase domains for the substrate translocation (Ilangovan et al. 2017).

Apart from these three-functional domains there is a fourth poorly characterized C-terminal domain. This domain consists of 295 residues (1461 to 1756) and is mostly flexible. It has been hypothesized to function as a platform for interaction with other relaxosomal proteins. Some studies also emphasize that the far C-terminal domain interacts with ds DNA (Matson and Ragonese 2005; Cheng et al. 2011; Ragonese et al. 2007).

There is no involvement of R1 TraI’s C-terminal domain on the regulation of its own nuclease and relaxase activity. A crystal structure of a 169 residue fold in the C-terminal domain (residues 1461 to 1630) has been reported however, structural and functional details of the far most C-terminal 126 residues (R1 TraIC126, residues 1630 to 1756, 14.3 kDa) are lacking. Due to the fact that this region could never be crystallized or observed in Cryo-EM, it was thought to be highly flexible and disordered (Ilangovan et al. 2017). An analysis of R1 TraIC126 (1630 to 1756) by the program COILS, predicted three coiled-coil domains (Guogas et al. 2009). R1 TraIC126 does not have an orthologue nor does it share sequence homology, thus this novel region appears to play a crucial role exclusively for R1 TraI. In this study, we present the ^1^H, ^13^C and ^15^N NMR resonance assignments and the secondary structure information obtained by Talos-N (Shen and Bax 2013) based on the backbone chemical shift values.

## Recombinant protein cloning, expression and purification

R1 TraIC126 was cloned into pET Z2 vector (EMBL Heidelberg) (Bogomolovas et al. 2009) by classical cloning methods where full length R1 TraI was used as a template. The two primers: R1 TraIC126-***F*** 5′-AGTCCATGGGCAATAGCGTCCAGCCG-3′ and R1 TraIC126-***R*** 5′-TTTGGTACCCTAATCGCCGCCCAA-3′, were used to create the R1 TraIC126 encoding insert by PCR amplification. pET Z2 contains a 6xHis, IgG binding domain (Z-domain), TEV cleavage site, followed by a multi-cloning site and carries a kanamycin resistance gene. Restriction digestion was carried out with NcoI and KpnI to create sticky ends for both the amplified R1 TraIC126 gene fragment and the template for subsequent ligation.

This pET Z2 R1 TraIC126 construct was transformed into BL21 (DE3) cells (Thermofisher, Waltham, US) and expressed in M9 media (containing 1 g/l ^15^N ammonium chloride and 3 g/l ^13^C glucose) for isotope labelling of proteins for NMR experiments (M9 minimal medium (standard) 2010). In 1 L of culture, the protein expression was induced with 1 mM (final concentration) of Isopropyl-β-D-thiogalactopyranoside (IPTG) at an OD_600_ nm of 0.8 and growth was continued with shaking at 20 °C for 16 h.

The cells were harvested and re-suspended in lysis buffer (50 mM Na_2_HPO_4_, 10 mM imidazole, 300 mM NaCl pH 8.0). Re-suspended cells were disrupted using sonication and centrifugation at 13,500 rpm was carried out to separate the cell pellet from the supernatant. The supernatant was filtered through a 0.45 µm filter and protein purification was performed using Ni–NTA affinity chromatography. An equilibrated 10 ml HisTrap column (GE Healthcare, Merck KGaA, Darmstadt, Germany) (with HisTrap flow through buffer: 50 mM Na_2_HPO_4_, 10 mM imidazole, 300 mM Nacl, pH 8.0 and HisTrap elution buffer: 50 mM Na_2_HPO_4_, 500 mM imidazole, 300 mM Nacl, pH 8.0) was loaded with 10 ml of filtered supernatant and the protein was eluted with HisTrap elution buffer, using an imidazole gradient. The eluted protein was subjected to an overnight TEV cleavage reaction. For this reaction, TEV protease (1:50 TEV protease to protein ratio), 4 mM β-mercaptoethanol was added to the sample and dialyzed against 2 L of dialysis buffer (50 mM Na_2_HPO_4_, 10 mM imidazole, 300 mM NaCl, 4 mM β-mercaptoethanol, pH 8.0) for 16 h. This step removes the 6xHIS and Z-domain. A second Ni–NTA purification separated the cleaved 6xHis and Z domain, eluting the protein during the flow through step. Final purification was done by size exclusion chromatography using a Superdex 75S column (GE Healthcare, Merck KGaA, Darmstadt, Germany) in 50 mM Na_2_HPO_4_, 300 mM Nacl, pH 8.0. The purified protein was dialyzed into the NMR buffer (50 mM Na_2_HPO_4_, 50 mM Nacl, pH 6.5) for 16 h, before acquiring the NMR experiments.

## NMR experiments

NMR measurements were carried out on a Bruker Avance III 700 MHz spectrometer equipped with a cryogenically cooled 5 mm TCI probe head. All the experiments were carried out at 298K. 10% D_2_O was added to the sample and the following triple resonance experiments were acquired for the assignment of the backbone atoms resonances: ^1^H–^15^N HSQC, ^1^H–^13^C HSQC, HNCACB, HNCA, HN(CA)CO, HNCO, HN(CO)CA and HNCANNH. For side-chain assignments, CC(CO)NH, H(CCO)NH, HBHA(CO)NH and HCCH-TOCSY were acquired. Complete assignments and confirmation thereof was accomplished using ^15^N-edited NOESY-HSQC and ^13^C-edited NOESY-HSQC (John et al. 2006). Standard Bruker pulses were used to acquire all the above mentioned triple resonance experiments. The spectra were processed using NMRPipe (Delaglio et al. 1995) and analyzed by CCPNmr (Vranken et al. 2005).

## Assignments and secondary structure information

**Fig. 2:**
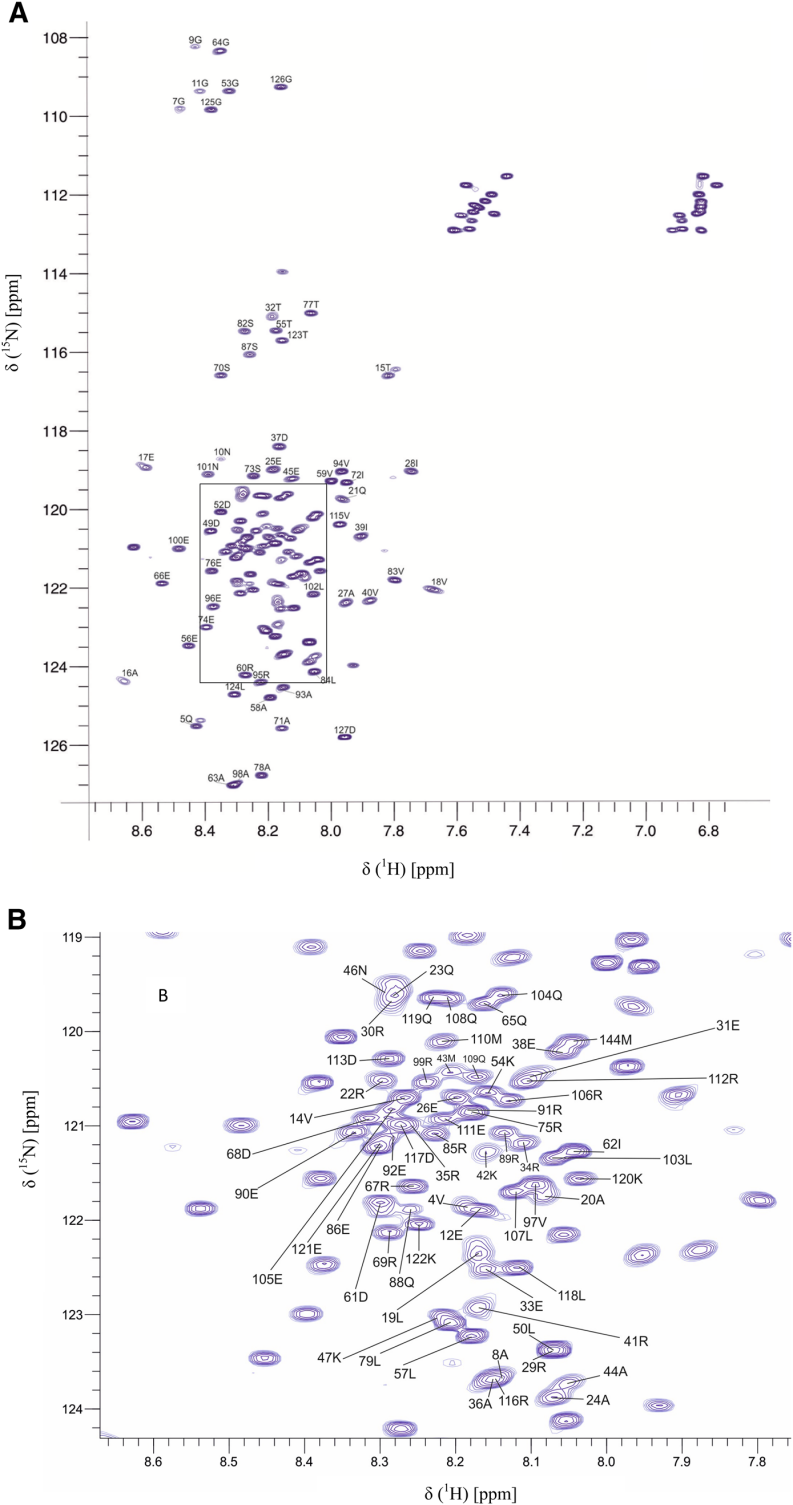
**a**^1^H–^15^N HSQC spectrum of R1 TraIC126 acquired at 700 MHz and pH 6.5 at 298K with 10% D_2_O. The number and the respective single letter code of amino acids are indicated at each peak. Residue numbers 2 to127 of R1 TraIC126 correspond to residues 1630 to 1756 in the full length TraI. Met1 is added during the cloning and is not a part of R1 TraIC126. **b** The zoomed region of the ^1^H–^15^N HSQC spectrum, highlighted in (**a**). BMRB access number 27,596

Figure 2a shows the ^1^H–^15^N HSQC spectrum of R1 TraIC126 and the apparent limited peak dispersion along the hydrogen dimension, all the backbone amide peaks between 7.6 and 8.7 ppm, shows that R1 TraIC126 is at least partially disordered. The most overlapped region of the HSQC is shown enlarged in Fig. 2b. With the help of the above mentioned backbone spectra, 94% of all ^1^H, ^15^N, ^13^C′, ^13^C^α/β^, 97% H^α^ and 93% H^β^ resonances were assigned. Asn2 was not found in the spectra. The amide nitrogen signals of the five proline residues along with Ser3 and Glu81 could not be assigned. Due to extensive line broadening or overlapping of peaks Arg30 C^β^, Arg34 C^β^, Arg35 C^β^, Pro6 C′, Pro80 C′ and the side chain amide of asparagine and glutamine residues were not assigned. The unassigned peaks in the ^15^N HSQC do not show correlations in the triple resonance experiments and therefore probably belong to the 6% of NH groups which could not be assigned.

**Fig. 3:**
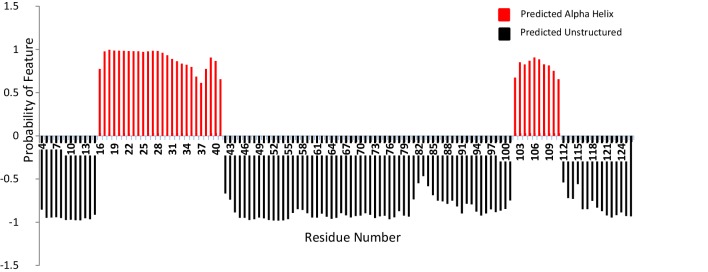
Secondary structure prediction of R1 TraIC126: The predictions from Talos-N are presented as bars colored in red for α helix and black for loop/disordered region. A negative score has been given for the loop region for the ease of comparison

Secondary structure information was obtained from the backbone chemical shifts processed using Talos-N. The prediction shows major disordered regions, as expected from the ^1^H–^15^N HSQC spectrum’s poor peak dispersion along the hydrogen dimension. Talos prediction shows that two stretches of helices, spanning from residues 16 to 41 and 102 to 111 are connected through a loop region. The residues 3 to 15, 42 to 101 and 112 to 127 are predicted to be loop / disordered regions (Fig. 3).
